# Design of Wideband Flextensional Hydrophone

**DOI:** 10.3390/s24154941

**Published:** 2024-07-30

**Authors:** Gihyeon Kim, Donghyun Kim, Yongrae Roh

**Affiliations:** School of Mechanical Engineering, Kyungpook National University, Daegu 41566, Republic of Korea; f12gto5800@naver.com (G.K.); roy4435@naver.com (D.K.)

**Keywords:** flextensional hydrophone, receiving voltage sensitivity (RVS), low frequency broadband hydrophone

## Abstract

Flextensional transducers have been widely used as low-frequency projectors, and these characteristics can be used to develop hydrophones with wider receiver bandwidth and higher sensitivity than conventional products in low-frequency ranges. In this work, we designed flextensional hydrophones of all classes, and compared their acoustic receiver performance to select the most suitable class for a low-frequency broadband hydrophone. For this purpose, basic models of the hydrophones were constructed for all classes and the effects of various structural parameters on the acoustic receiver characteristics of the hydrophones were analyzed. Based on the results, the structure of the flextensional hydrophone of each class was designed to have the maximum receiver bandwidth by an optimization technique while maintaining the receiver voltage sensitivity over a certain level. A comparison of the designed performance led to the selection of the class IV flextensional hydrophone as the most promising one with the widest receiver fractional bandwidth and highest sensitivity.

## 1. Introduction

Hydrophones are devices that detect underwater sound waves [1,2]. Low-frequency acoustic signals have relatively long wavelengths and low attenuation, making them essential for communication systems in open oceans [3]. Since many underwater vehicles, such as submarines and multi-purpose unmanned underwater vehicles, operate in deep and large marine areas, hydrophones with excellent sensitivity to low-frequency underwater acoustic signals are required to detect them [4]. Common piezoelectric hydrophones include spherical hydrophones, ring hydrophones, and cylindrical hydrophones, which are widely used in marine life research, underwater seismic monitoring, and ship and submarine detection [1,3,5]. However, their use for oceanic area detection is limited because their reception characteristics are not flat in the low-frequency range [5].

Flextensional transducers have been studied since the 1950s and are now frequently used as low-frequency projectors for underwater detection and communication. They are classified into seven classes based on their shape and operating principle [6]. Starting with classes I and IV, there is class II that extends the length of the piezoceramic stack to increase the transmission power of class I, and class III that joins two shells with different radii of curvature to expand the transmitting bandwidth of class I [7,8]. In addition, there is class V that utilizes the radial vibration mode of a piezoceramic disk, unlike typical flextensional transducers that make use of the thickness vibration mode [9]. Class VII and class VI are modifications of class IV and class V, respectively, to have concave shells for deep water use [10,11,12].

In recent research on flextensional transducers, Somayajula et al. used piezoceramics with a high piezoelectric charge constant to achieve high transmitting power in the first flexural mode of a class I transducer [13]. To increase the transmitting bandwidth, Hladky-Hennion et al. proposed a wagon wheel structure, which is a variation of the class I transducer, and Li et al. proposed a negative curvature shell structure, which is a variation of the class IV shell [14,15]. Zhou et al. proposed a structure with a class IV piezoceramic stack attached to the shell to increase the transmitting power and bandwidth [16]. A cymbal transducer has been used as a miniaturized version of the class V, and much research has been carried out to develop structures to increase transmitting power and bandwidth [17,18,19]. However, these are all studies using flextensional transducers as projectors and there have been very few works on flextensional transducers being used as hydrophones.

As examples of studies using flextensional transducers as hydrophones, Sun et al. used brass and PZT-5A as the cap and piezoceramic material of the cymbal hydrophone, and proposed a new structure to increase the peak receiving voltage sensitivity (RVS) level and to lower the resonance frequency [20]. Lonkar et al. used PNS-PZT, which has a high piezoelectric charge constant, to increase the peak RVS level of a cymbal hydrophone and compared the RVS with that of another cymbal using PZT-5A [21]. By varying two structural parameters, piezoceramic disk diameter and cavity depth, Kannan et al. developed a wideband cymbal hydrophone structure [22]. Recently, Kim and Roh reported the new development of cymbal hydrophones for broadband [23] and vector hydrophone applications. Although previous research using flextensional transducers as projectors has used a variety of classes, precedent research using them as hydrophones has been limited to cymbal transducers, and no studies have compared the receiver characteristics of different classes of flextensional transducers. Very recently, the authors of this paper presented a preliminary comparison [24]. In addition, although high sensitivity and broadband receive characteristics, especially in the low-frequency range, are important for use as oceanic area hydrophones, there have been few studies on the systematic analysis and structural design of flextensional hydrophones to achieve these properties.

Therefore, in this study, we developed a flextensional hydrophone with a wide frequency bandwidth and high RVS in the low-frequency range. The low-frequency range referred to in this study spans from several Hz to several kHz. Since flextensional transducers are often used as low-frequency projectors, using flextensional transducers as hydrophones would compensate for the narrow operating frequency range and low sensitivity of conventional hydrophones in the low-frequency range [1,6,25]. Further, flextensional hydrophones can be designed to detect submarines or unmanned underwater vehicles that typically operate at depths of tens to hundreds of meters. For this purpose, first, we built basic models of flextensional transducers of all of the classes whose peak RVS frequency fell within a certain range, and analyzed the effects of various structural parameters on the acoustic receiver characteristics of the transducers. Then, through optimization, we derived the structure of each class with the widest bandwidth in the low-frequency range and the highest RVS. By comparing the RVS spectra of all of the classes, we selected the class that had the highest performance as a hydrophone in terms of the receiver bandwidth and the RVS level at a low frequency. Finally, the characteristics of the designed hydrophone were compared with those of existing representative commercial hydrophones. The simulated performance of the designed hydrophone is expected to surpass that of existing models, being positioned as a superior low-frequency broadband hydrophone.

## 2. Structure of the Flextensional Hydrophones

### Construction of Finite Element Analysis Models

**Figure 1:**
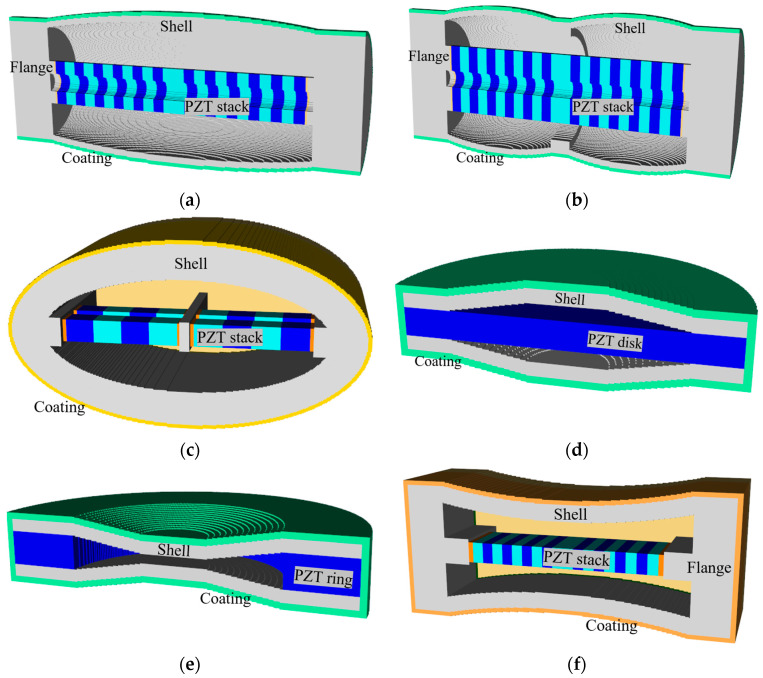
Flextensional hydrophones: (**a**) class I, (**b**) class III, (**c**) class IV, (**d**) class V, (**e**) class VI, and (**f**) class VII.

In this study, basic models of all classes of flextensional transducers were constructed using PZFlex^®^ (version 2018), a commercial finite element analysis (FEA) program, and their acoustic properties were analyzed. Figure 1 shows the basic model for each class. The bar in the middle of the transducers in Figure 1a–d represents the drive section, which consists of multiple piezoelectric disks with adjacent layers stacked in opposite polarization directions. In these figures, disks of different colors indicate different polarization directions. Conversely, the class V and VI transducers feature a piezoceramic disk with a single polarization direction at the center of their structures. The models were surrounded with water, large enough to account for an acoustic far field. An absorbing boundary condition was enforced on the edges of the water to prevent the reflection of sound waves. The meshed models of the underwater hydrophones were composed of 0.5 mm elements. The element size in the model was determined through a convergence test to ensure accuracy. The number of elements amounted to 228 million and the number of nodes was 226 million for the class IV model. To calculate the RVS, a plane wave of 1 Pa of an impulsive form was radiated from a plane source located at a far field distance towards the hydrophone. Then, the voltage generated at the hydrophone and the sound pressure applied to the position of the hydrophone were calculated and substituted into Equation (1) as *V*_o_ and *P*_i_, respectively. The response of the hydrophones to the incident plane wave was simulated through transient analyses. The output voltages from the hydrophone were then transformed into the frequency domain using the Fast Fourier Transform, enabling a detailed examination of the hydrophone’s behavior. All of the hydrophone classes used PZT-4 as the piezoceramic, rubber as the coating material, aluminum as the shell material, and steel as the end plate material, whose properties are displayed in Table 1 [26].

(1) $$RVS=20\log\left(V_{out}/P_{i}\right)\left(dB\;re\;1\;V/\mu Pa\right)$$

**Figure 2:**
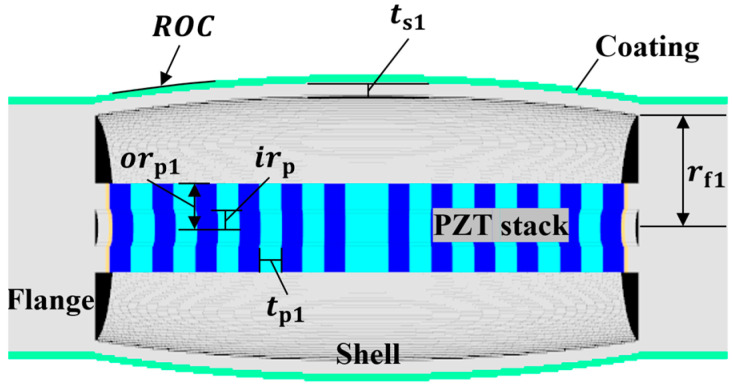
Class I flextensional hydrophone model, where *t*_s1_ is the shell thickness, $r_{f1}$ is the flange radius, *ROC* is the radius of the curvature of the shell, $t_{p1}$ is the piezoceramic disk thickness, ${or}_{p1}$ is the outer radius of the piezoceramic disk, and ${ir}_{p}$ is the inner radius of the piezoceramic disk. Refer to Table 2 for detailed dimensions of the parameters.

**Figure 3:**
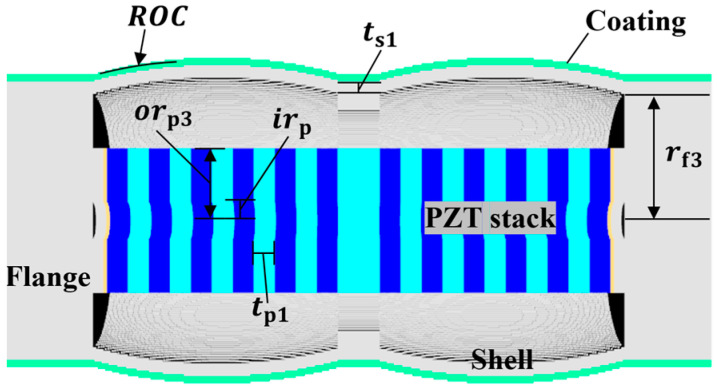
Class III flextensional hydrophone model, where $r_{f3}$ is the flange radius and ${or}_{p3}$ is the outer radius of the piezoceramic disk. The other parameters are the same as those in Figure 2. Refer to Table 3 for detailed dimensions of the parameters.

**Figure 4:**
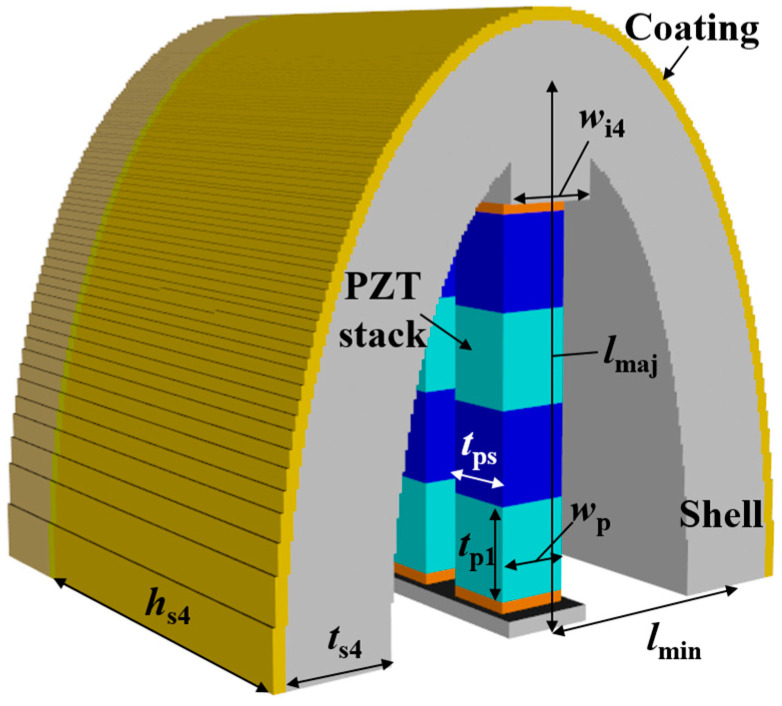
Class IV flextensional hydrophone model, where *t*_s4_ is the shell thickness, *h*_s4_ is the shell height, $l_{min}$ is the semi minor axis length, $l_{maj}$ is the semi major axis length, $t_{ps}$ is the piezoceramic stack thickness, $w_{i4}$ is the insert width, and $w_{p}$ is the piezoceramic stack width. Refer to Table 4 for detailed dimensions of the parameters.

**Figure 5:**
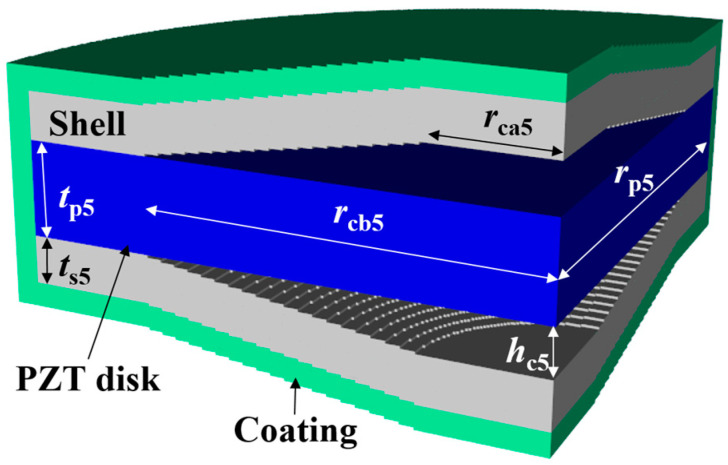
Class V flextensional hydrophone model, where $r_{ca5}$ is the cavity apex radius, $r_{cb5}$ is the cavity base radius, $r_{p5}$ is the piezoceramic disk radius, $h_{c5}$ is the cavity height, $t_{p5}$ is the piezoceramic disk thickness, $t_{s5}$ is the shell thickness, and $r_{ca5}$ is the cavity apex radius. Refer to Table 5 for detailed dimensions of the parameters.

**Figure 6:**
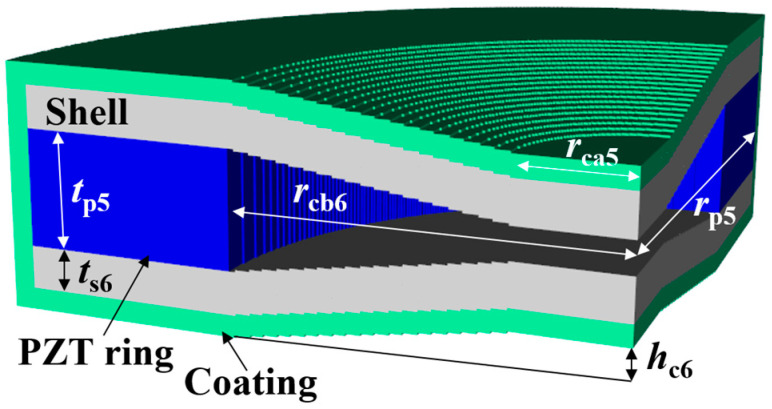
Class VI flextensional hydrophone model, where $r_{cb6}$ is the cavity base radius, $h_{c6}$ is the cavity height, and $t_{s6}$ is the shell thickness. The other parameters are the same as those in Figure 5. Refer to Table 6 for detailed dimensions of the parameters.

**Figure 7:**
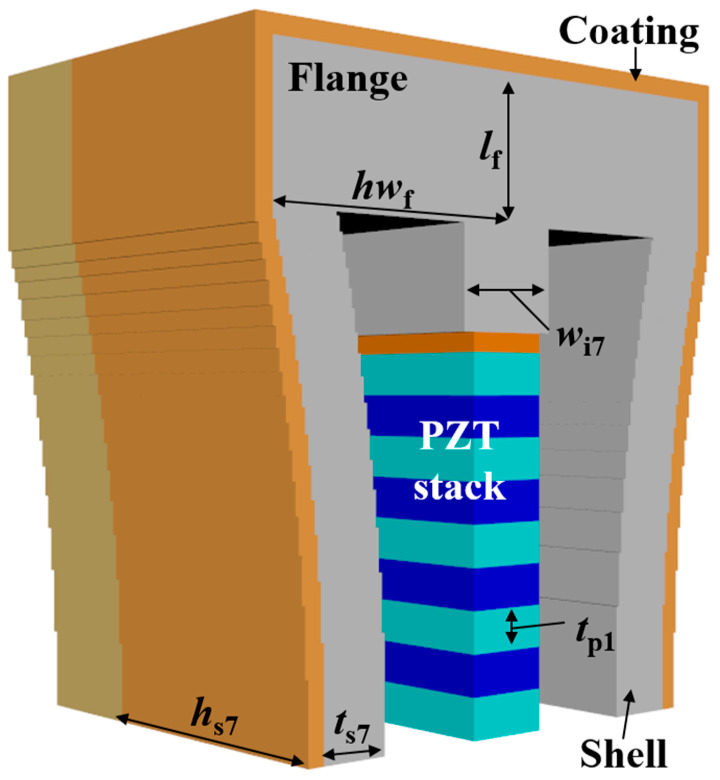
Class VII flextensional hydrophone model, where *t*_s7_ is the shell thickness, *h*_s7_ is the shell height, ${hw}_{f}$ is the flange half-width, $l_{f}$ is the flange length, and $w_{i7}$ is the insert width. Refer to Table 7 for detailed dimensions of the parameters.

Class II is an extension of the piezoceramic stack length of class I to increase transmitting power, which is not related to the receiver characteristics, so was excluded from the analysis of this study. Also, as mentioned in the introduction, the concave-type flextensional transducers were developed for use in deep water, not to improve the acoustic characteristics. Therefore, only the convex types were used as the transducers for classes I and III in this study. Classes I and III are axially symmetrical with respect to the piezoceramic stack and aluminum shell, as shown in Figure 2 and Figure 3. The dimensions of the basic class I and III models are shown in Table 2 and Table 3, respectively. The class III dimensions not shown in Table 3 are the same as those in Table 2. These dimensions were roughly determined by preliminary analysis to ensure that the hydrophones of all of the classes had a peak RVS frequency within 8–10 kHz. Class IV has a piezoceramic stack enclosed in an oval shell, as shown in Figure 4, with end plates attached to the shell to prevent water ingress. Table 4 shows the basic dimensions of this model, with *t*_p1_ equal to the value in Table 2. Classes V and VI consist of cymbal-like caps attached to the top and bottom of a piezoceramic disk, as shown in Figure 5 and Figure 6. The dimensions of these basic models are presented in Table 5 and Table 6. The class VI dimensions not shown in Table 6 are the same as those in Table 5. Class VII has the same structure as that of the class IV, except that the shell has a concave shape and flanges are attached to the ends of the piezoceramic stack, as shown in Figure 7. The dimensions of its basic model are shown in Table 7, with *t*_p1_ equal to that in Table 2.

## 3. Effects of Structural Parameters on the Receiver Characteristics of Flextensional Hydrophones

Prior to rigorously designing the flextensional hydrophones, the impact of each structural parameter on the acoustic properties of the hydrophones was analyzed with the models constructed in Section 2, and the structural parameters with the greatest impact were selected as design variables with which to carry out further design. Three acoustic characteristics were selected as comparison criteria: receiver frequency bandwidth, RVS at 100 Hz, and peak RVS frequency. The receiver frequency bandwidth was defined as the range up to the frequency at which the RVS level differed from that at 100 Hz by 3 dB. Since this study is interested in the low-frequency range, the RVS level at 100 Hz was chosen as the reference frequency with which to define the receiver bandwidth. Normally, an RVS spectrum maintains a constant level in the low-frequency range and then increases as the frequency approaches the peak RVS frequency. This trend indicates that the peak RVS frequency is a significant factor in determining the bandwidth. Therefore, the peak RVS frequency was also chosen as a comparative acoustic characteristic because a constant peak RVS frequency was required to make a fair comparison between the different classes of flextensional hydrophones.

### 3.1. Class I and III Flextensional Hydrophones

**Figure 8:**
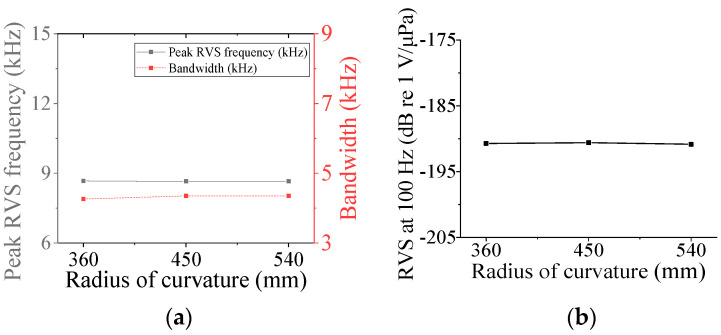
Variation in acoustic characteristics of the class I flextensional hydrophone according to the change in *ROC*: (**a**) peak RVS frequency and bandwidth, (**b**) RVS at 100 Hz.

**Figure 9:**
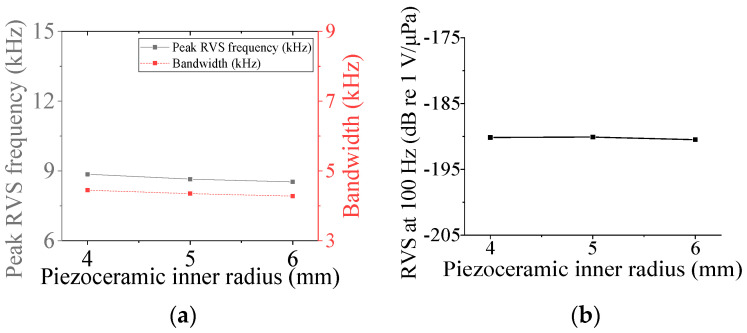
Variation in acoustic characteristics of the class I flextensional hydrophone according to the change in *ir*_p_: (**a**) peak RVS frequency and bandwidth, (**b**) RVS at 100 Hz.

**Figure 10:**
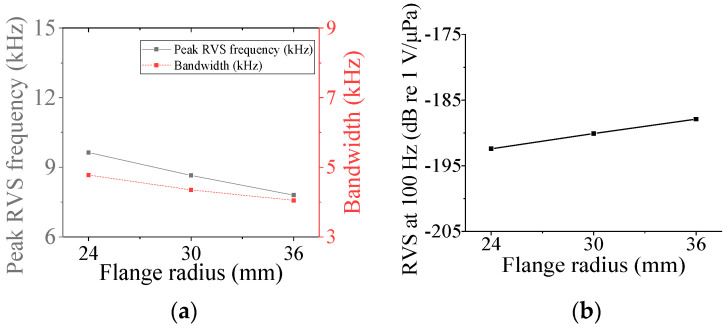
Variation in acoustic characteristics of the class I flextensional hydrophone according to the change in *r*_f1_: (**a**) peak RVS frequency and bandwidth, (**b**) RVS at 100 Hz.

**Figure 11:**
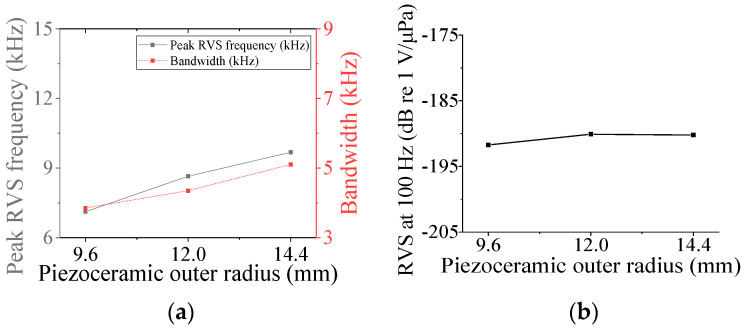
Variation in acoustic characteristics of the class I flextensional hydrophone according to the change in *or*_p1_: (**a**) peak RVS frequency and bandwidth, (**b**) RVS at 100 Hz.

**Figure 12:**
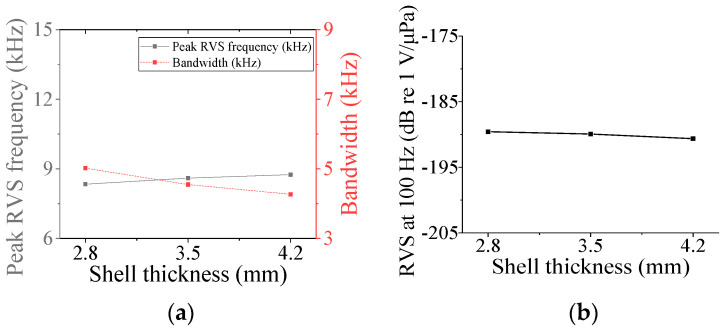
Variation in acoustic characteristics of the class I flextensional hydrophone according to the change in *t*_s1_: (**a**) peak RVS frequency and bandwidth, (**b**) RVS at 100 Hz.

First, the acoustic characteristics of the class I flextensional hydrophone were analyzed according to the changes in its structural parameters: the radius of curvature (*ROC*), shell thickness (*t*_s1_), flange radius (*r*_f1_), and inner radius (*ir*_p_) and outer radius (*or*_p1_) of the piezoceramic disk. The results are shown in Figure 8, Figure 9, Figure 10, Figure 11 and Figure 12 [27]. The range of variation for each parameter was set to ±20% from the basic dimension. As seen in Figure 8 and Figure 9, changes in *ROC* and *ir*_p_ turned out to have little effect on the acoustic characteristics of the hydrophone. As shown in Figure 10, as *r*_f1_ became longer, the RVS at 100 Hz increased, while the peak RVS frequency and bandwidth tended to decrease. *or*_p1_ tended to increase the peak RVS frequency and bandwidth as it became longer, but the RVS at 100 Hz had the highest value when *or*_p1_ was the basic dimension, contrary to the effect of *r*_f1_. As seen in Figure 12, as *t*_s1_ became thicker, the peak RVS frequency increased, but the bandwidth tended to decrease. This showed that *t*_s1_ was an important parameter to increase the bandwidth while maintaining the peak RVS frequency constant. Based on these results, *t*_s1_, *r*_f1_, and *or*_p1_ were selected as design variables with a large influence on the acoustic characteristics for the class I hydrophone. For the class III hydrophone, the tendency of acoustic characteristics variation in relation to the change in its structural parameters was almost the same as that of the class I hydrophone, so *t*_s1_, *r*_f3_, and *or*_p3_ were selected as design variables as well.

### 3.2. Class IV Flextensional Hydrophone

**Figure 13:**
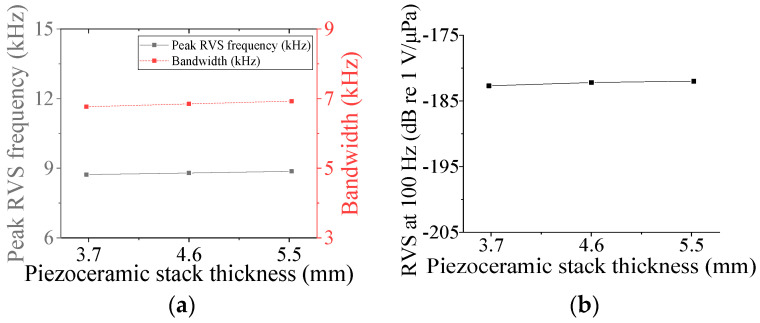
Variation in acoustic characteristics of the class IV flextensional hydrophone according to the change in *t*_ps_: (**a**) peak RVS frequency and bandwidth, (**b**) RVS at 100 Hz.

**Figure 14:**
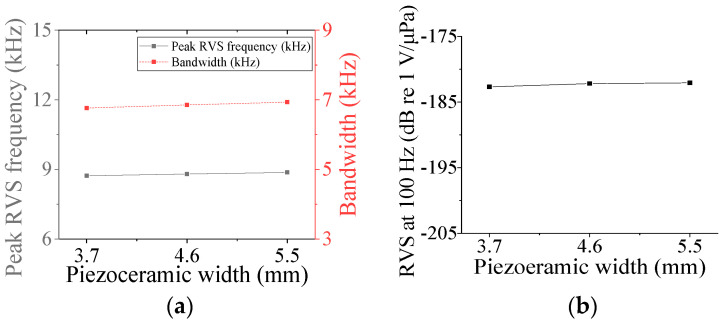
Variation in acoustic characteristics of the class IV flextensional hydrophone according to the change in *w*_p_: (**a**) peak RVS frequency and bandwidth, (**b**) RVS at 100 Hz.

**Figure 15:**
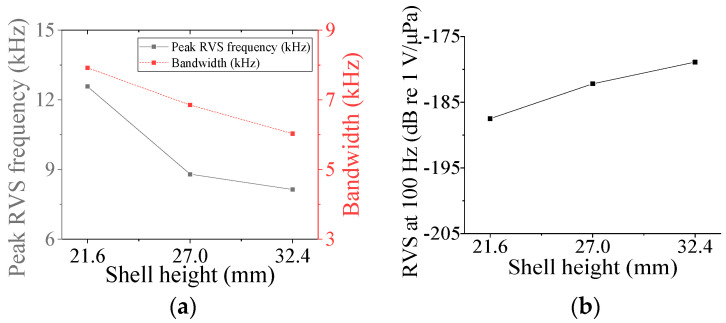
Variation in acoustic characteristics of the class IV flextensional hydrophone according to the change in *h*_s4_: (**a**) peak RVS frequency and bandwidth, (**b**) RVS at 100 Hz.

**Figure 16:**
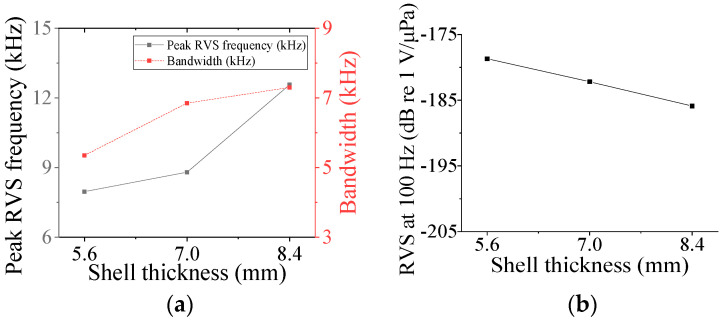
Variation in acoustic characteristics of the class IV flextensional hydrophone according to the change in *t*_s4_: (**a**) peak RVS frequency and bandwidth, (**b**) RVS at 100 Hz.

**Figure 17:**
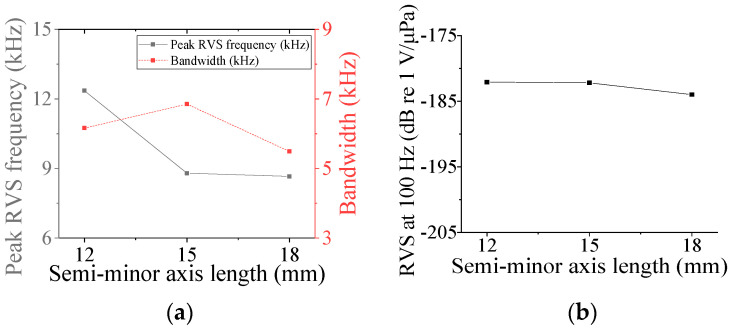
Variation in acoustic characteristics of the class IV flextensional hydrophone according to the change in *l*_min_: (**a**) peak RVS frequency and bandwidth, (**b**) RVS at 100 Hz.

Five parameters were used to analyze the performance variation of the class IV flextensional hydrophone: shell thickness (*t*_s4_), shell height (*h*_s4_), semi minor axis length (*l*_min_), piezoceramic stack thickness (*t*_ps_), and piezoceramic stack width (*w*_p_) [28]. Variations in the acoustic characteristics of the hydrophone analyzed by increasing or decreasing the basic dimensions of the parameters by 20% are presented in Figure 13, Figure 14, Figure 15, Figure 16 and Figure 17. As can be seen in Figure 13 and Figure 14, *t*_ps_ and *w*_p_ had little effect on the acoustic characteristics of the hydrophone. According to Figure 15, as *h*_s4_ increased, the RVS at 100 Hz increased, while the peak RVS frequency and bandwidth tended to decrease. Figure 16 shows that as *h*_s4_ became thicker, the RVS at 100 Hz decreased, while the peak RVS frequency and bandwidth tended to increase. However, as seen in Figure 17, *l*_min_ showed that the peak RVS frequency was constant at the basic dimension (15 mm) and the maximum dimension (18 mm), while the bandwidth tended to widen as it approached the basic dimension. The *l*_min_ played a role structurally similar to that of the *ROC* of the class I transducer, but had a significant effect on the peak RVS frequency and bandwidth. The reason for this is that a class IV transducer does not have a flange structure, but instead has a shell that acts as a flange. Lengthening the *l*_min_ of the class IV transducer had the same effect as shortening the *ROC* of the class I transducer and lengthening *r*_f1_, which seemed to have a significant effect on the stiffness of the hydrophone. Based on the above results, *t*_s4_, *h*_s4_, and *l*_min_ were selected as the design variables for the class IV hydrophone.

### 3.3. Class V and VI Flextensional Hydrophone

**Figure 18:**
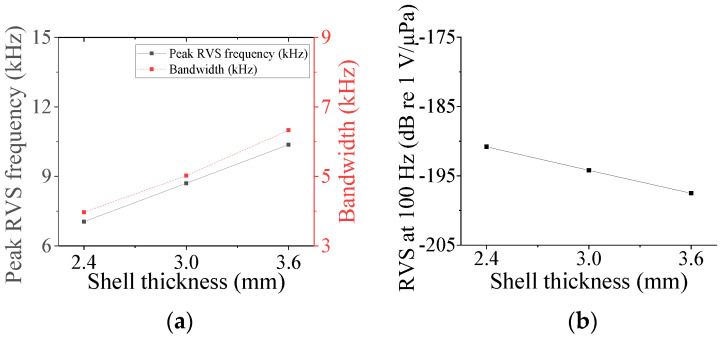
Variation in acoustic characteristics of the class V flextensional hydrophone according to the change in *t*_s5_: (**a**) peak RVS frequency and bandwidth, (**b**) RVS at 100 Hz.

**Figure 19:**
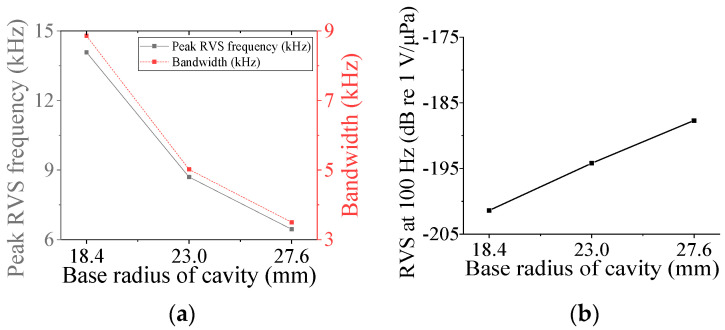
Variation in acoustic characteristics of the class V flextensional hydrophone according to the change in *r*_cb5_: (**a**) peak RVS frequency and bandwidth, (**b**) RVS at 100 Hz.

**Figure 20:**
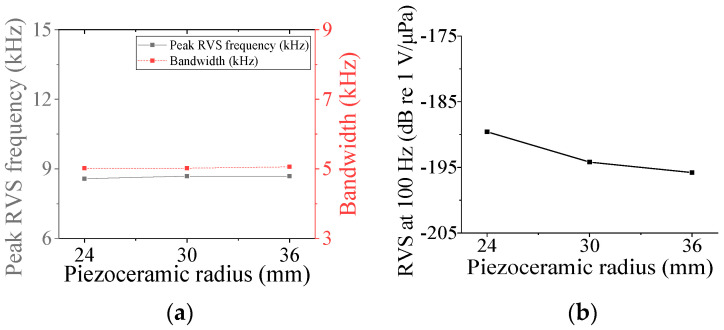
Variation in acoustic characteristics of the class V flextensional hydrophone according to the change in *r*_p5_: (**a**) peak RVS frequency and bandwidth, (**b**) RVS at 100 Hz.

**Figure 21:**
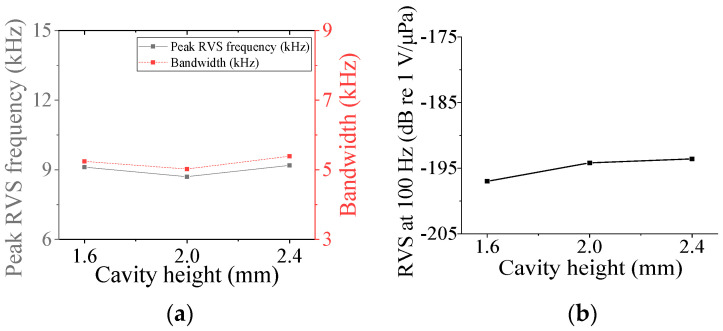
Variation in acoustic characteristics of the class V flextensional hydrophone according to the change in *h*_c5_: (**a**) peak RVS frequency and bandwidth, (**b**) RVS at 100 Hz.

Four parameters were selected for characterization of the class V flextensional hydrophone: cavity base radius (*r*_cb5_), piezoceramic disk radius (*r*_p5_), shell thickness (*t*_s5_), and cavity height (*h*_c5_). Figure 18, Figure 19, Figure 20 and Figure 21 show changes in the acoustic characteristics of the hydrophone when the size of each parameter was increased or decreased by 20% from the basic size. In Figure 18, as *t*_s5_ became thicker, the RVS at 100 Hz decreased, while peak RVS frequency and bandwidth tended to increase. As seen in Figure 19, as r_cb5_ became longer, the RVS at 100 Hz increased, while the peak RVS frequency and bandwidth tended to decrease. *r*_cb5_ appeared to have a significant effect on the stiffness of the hydrophone by changing the contact area between the shell and the piezoceramic disk. Figure 20 shows that *r*_p5_ had a significant effect on the RVS at 100 Hz, but had little effect on bandwidth, which is the main concern of this study, so it was excluded from the design variables. For the same reason, *h*_c5_ was selected as a design variable because its effect on bandwidth was not negligible, as shown in Figure 21. Similar to the case of class I and III hydrophones, the variation in acoustic characteristics of the class VI hydrophone in relation to the change in its structural parameters was almost the same as that in the class V hydrophone, so the selected design variables were the same, i.e., *t*_s6_, *h*_c6_, and *r*_cb6_.

### 3.4. Class VII Flextensional Hydrophone

**Figure 22:**
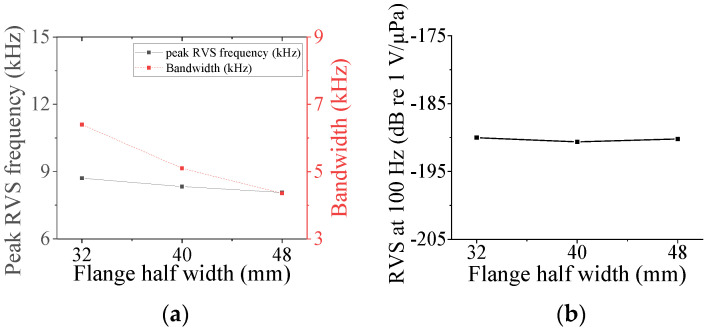
Variation in acoustic characteristics of the class VII flextensional hydrophone according to the change in *hw*_f_: (**a**) peak RVS frequency and bandwidth, (**b**) RVS at 100 Hz.

**Figure 23:**
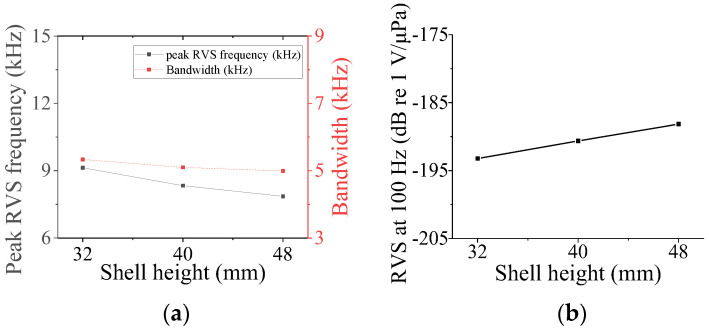
Variation in acoustic characteristics of the class VII flextensional hydrophone according to the change in *h*_s7_: (**a**) peak RVS frequency and bandwidth, (**b**) RVS at 100 Hz.

**Figure 24:**
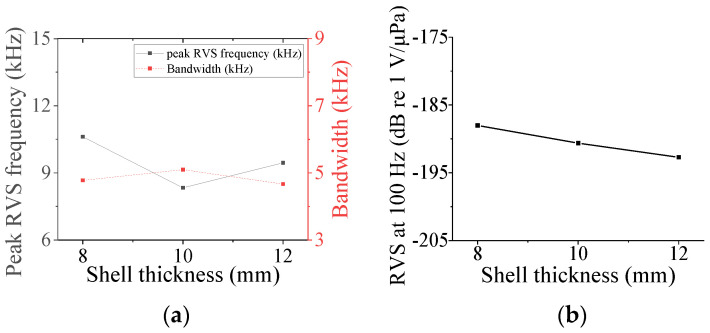
Variation in acoustic characteristics of the class VII flextensional hydrophone according to the change in *t*_s7_: (**a**) peak RVS frequency and bandwidth, (**b**) RVS at 100 Hz.

**Figure 25:**
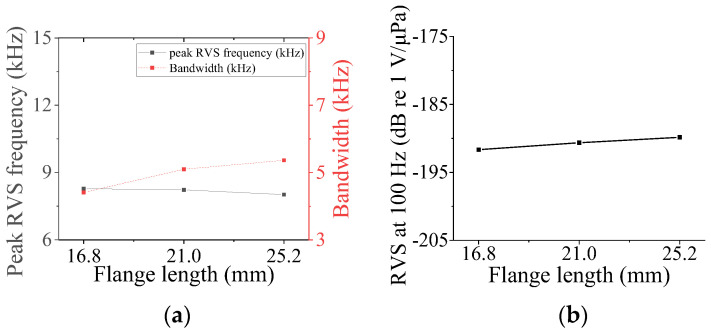
Variation in acoustic characteristics of the class VII flextensional hydrophone according to the change in *l*_f_: (**a**) peak RVS frequency and bandwidth, (**b**) RVS at 100 Hz.

Four parameters were used to analyze the acoustic characteristics of the class VII flextensional hydrophone: shell thickness (*t*_s7_), shell height (*h*_s7_), flange length (*l*_f_), and flange half-width (*hw*_f_) [29]. These parameters were varied by 20% from their base dimensions, as shown in Figure 22, Figure 23, Figure 24 and Figure 25. Figure 22 and Figure 23 show that the peak RVS frequency and bandwidth decreased, but the RVS at 100 Hz increased as *hw*_f_ and *h*_s7_ became longer. As seen in Figure 24, as *t*_s7_ became thicker, the RVS at 100 Hz decreased, but the peak RVS frequency and the bandwidth exhibited their minimum and maximum at the basic dimension, respectively. Also, in Figure 25, the RVS at 100 Hz and bandwidth increased as the *l*_f_ increased, while the peak RVS frequency did not change significantly. This shows that *l*_f_ is an important parameter that can increase bandwidth while keeping the peak RVS frequency constant. From the above results, we selected *t*_s7_, *h*_s7_, *l*_f_, and *hw*_f_ as design variables of the class VII hydrophone.

## 4. Design of Broadband Flextensional Hydrophone Structures

**Figure 26:**
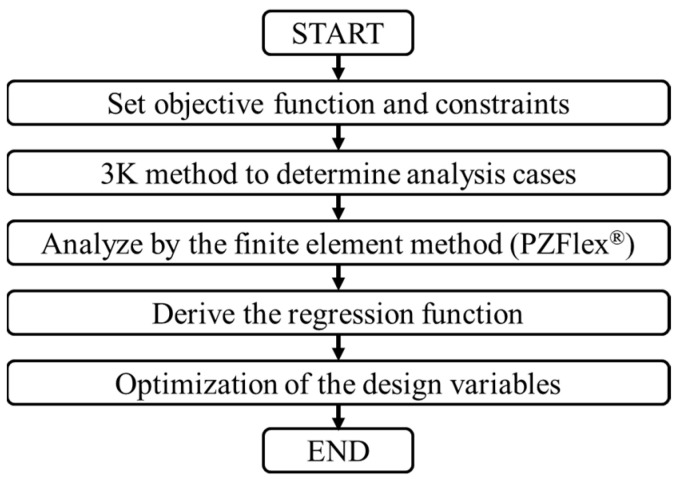
Optimal design process for a flextensional hydrophone of each class.

The structure of a broadband flextensional hydrophone of each class was designed using the design variables selected in Section 3. The optimal structure design scheme is presented in Figure 26. The same procedure was carried out repeatedly to design the structure of a broadband hydrophone of each class.

To begin with, as shown in Equation (2), an objective function was set up to maximize the bandwidth while satisfying the constraints that the peak RVS frequency should be 8.5 kHz with a tolerance of ±0.2 kHz, and the RVS at 100 Hz should be greater than that of the basic model of that class. The desired peak RVS frequency was arbitrarily set to 8.5 kHz by the authors, but it can be set to a different value depending on an application.

(2) $$\begin{array}{c} \mathrm{Objective}\;\mathrm{function}:\;\mathrm{minimize}\;(1/\mathrm{bandwidth})\\ \mathrm{Constraints}:\;1.\;8.3\;\mathrm{kHz}\;\leq \;\mathrm{Peak}\;\mathrm{RVS}\;\mathrm{frequency}\;\leq \;8.7\;\mathrm{kHz}\\ \;2.\;\mathrm{RVS}\;\mathrm{at}\;100\;\mathrm{Hz}\;\mathrm{of}\;\mathrm{the}\;\mathrm{model}\;\geq \;\mathrm{RVS}\;\mathrm{at}\;100\;\mathrm{Hz}\;\mathrm{of}\;\mathrm{the}\;\mathrm{basic}\;\mathrm{model} \end{array}$$

**Figure 27:**
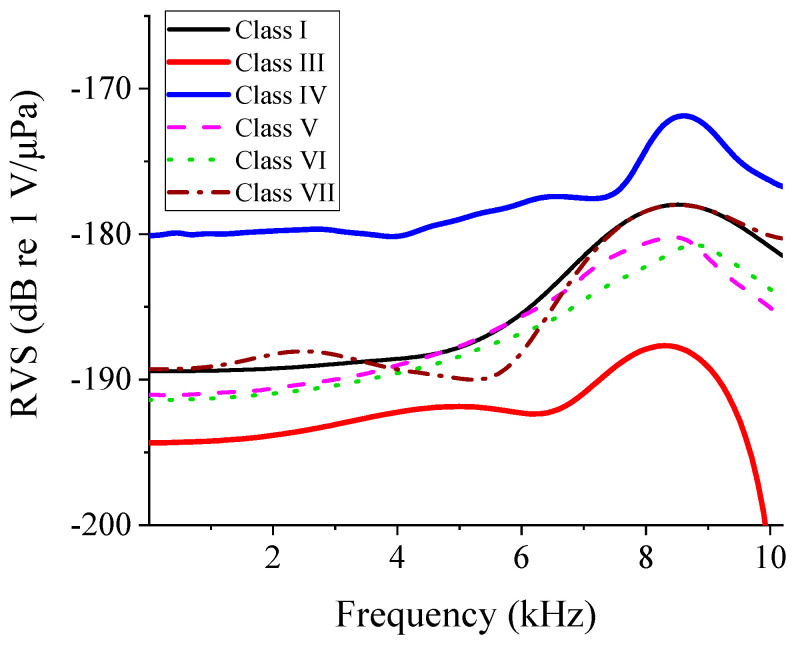
RVS spectra of the optimized flextensional hydrophone models of different classes.

The cases to be analyzed for optimization were selected using the 3k experimental design method [30]. The variation in the performance of the hydrophone, that is, bandwidth, peak RVS frequency, and RVS at 100 Hz, was collected in relation to the changes in design variables through FEA of the selected cases. Regression analysis was conducted on the collected data, and the hydrophone performance was derived as regression functions [31]. As an example, the regression functions derived for ‘Peak RVS frequency’, ‘RVS at 100 Hz’, and ‘Bandwidth’ of the class I hydrophone are shown in Equations (3)–(5), where x_1_ = *t*_s1_, x_2_ = *or*_p1_, and x_3_ = *r*_f1_. The regression functions are generalized functions valid only within the frequency range analyzed in this study, specifically from low Hz to 10 kHz, as shown in Figure 27. With the derived regression functions, the optimal combination of x_1_, x_2_, and x_3_ that could minimize the objective function while satisfying the constraints was obtained using the OptQuest Nonlinear Programming (OQNLP) algorithm [32]. The optimal values of the design variables to realize the broadband flextensional hydrophone obtained in this manner for each class are summarized in Table 8.

(3) $$\begin{array}{c} \mathrm{Bandwidth}\\ =\;0.0004650\;{x_{1}}^{2}\;{x_{2}}^{2}\;{x_{3}}^{2}\;-\;0.0003046\;{x_{1}}^{2}\;{x_{2}}^{2}\;{x_{3}}^{1}\;-\;0.0006253\;{x_{1}}^{2}\;{x_{2}}^{2}\;-\;0.0009484\;{x_{1}}^{2}\;{x_{2}}^{1}\;{x_{3}}^{2}\;-\;\\ \;0.0002810\;{x_{1}}^{2}\;{x_{2}}^{1}\;{x_{3}}^{1}\;+\;0.001225\;{x_{1}}^{2}\;{x_{2}}^{1}\;+\;0.0007988\;{x_{1}}^{2}\;{x_{3}}^{2}\;+\;0.0005685\;{x_{1}}^{2}\;{x_{3}}^{1}\;+\;0.001313\;\\ \;{x_{1}}^{2}\;-\;0.0005561\;{x_{1}}^{1}\;{x_{2}}^{2}\;{x_{3}}^{2}\;+\;0.0002075\;{x_{1}}^{1}\;{x_{2}}^{2}\;{x_{3}}^{1}\;+\;0.0004713\;{x_{1}}^{1}\;{x_{2}}^{2}\;+\;0.0009402\;{x_{1}}^{1}\;{x_{2}}^{1}\;\\ \;{x_{3}}^{2}\;-\;0.00004791\;{x_{1}}^{1}\;{x_{2}}^{1}\;{x_{3}}^{1}\;-\;0.001688\;{x_{1}}^{1}\;{x_{2}}^{1}\;+\;0.00005820\;{x_{1}}^{1}\;{x_{3}}^{2}\;-\;0.001590\;{x_{1}}^{1}\;{x_{3}}^{1}\;-\;\\ \;0.002696\;{x_{1}}^{1}\;+\;0.00001897\;{x_{2}}^{2}\;{x_{3}}^{2}\;+\;0.0001615\;{x_{2}}^{2}\;{x_{3}}^{1}\;+\;0.0002460\;{x_{2}}^{2}\;+\;0.00006823\;{x_{2}}^{1}\;\\ \;{x_{3}}^{2}\;+\;0.0002857\;{x_{2}}^{1}\;{x_{3}}^{1}\;+\;0.0006747\;{x_{2}}^{1}\;+\;0.00002366\;{x_{3}}^{2}\;+\;0.0004650\;{x_{3}}^{1}\;+\;0.02777 \end{array}$$

(4) $$\begin{array}{c} \mathrm{Peak}\;\mathrm{RVS}\;\mathrm{frequency}\\ =\;-327.01\;{x_{1}}^{2}\;{x_{2}}^{2}\;{x_{3}}^{2}\;-\;280.29\;{x_{1}}^{2}\;{x_{2}}^{2}\;{x_{3}}^{1}\;+\;23.36\;{x_{1}}^{2}\;{x_{2}}^{2}\;-\;116.79\;{x_{1}}^{2}\;{x_{2}}^{1}\;{x_{3}}^{2}\;-\;256.93\;{x_{1}}^{2}\;{x_{2}}^{1}\;\\ \;{x_{3}}^{1}\;-\;116.79\;{x_{1}}^{2}\;{x_{2}}^{1}\;-\;23.36\;{x_{1}}^{2}\;{x_{3}}^{2}\;+\;70.07\;{x_{1}}^{2}\;{x_{3}}^{1}\;+\;140.15\;{x_{1}}^{2}\;+\;280.29\;{x_{1}}^{1}\;{x_{2}}^{2}\;{x_{3}}^{2}\;+\;233.58\;\\ \;{x_{1}}^{1}\;{x_{2}}^{2}\;{x_{3}}^{1}\;-\;70.07\;{x_{1}}^{1}\;{x_{2}}^{2}\;+\;163.50\;{x_{1}}^{1}\;{x_{2}}^{1}\;{x_{3}}^{2}\;+\;210.22\;{x_{1}}^{1}\;{x_{2}}^{1}\;{x_{3}}^{1}\;+\;70.07\;{x_{1}}^{1}\;{x_{2}}^{1}\;-\;23.36\;{x_{1}}^{1}\;\\ \;{x_{3}}^{2}\;-\;116.79\;{x_{1}}^{1}\;{x_{3}}^{1}\;+\;420.44\;{x_{1}}^{1}\;+\;23.36\;{x_{2}}^{2}\;{x_{3}}^{2}\;+\;23.36\;{x_{2}}^{2}\;{x_{3}}^{1}\;-\;0.0005500\;{x_{2}}^{2}\;-\;23.36\;{x_{2}}^{1}\;\\ \;{x_{3}}^{2}\;+\;70.07\;{x_{2}}^{1}\;{x_{3}}^{2}\;-\;93.43\;{x_{2}}^{1}\;+\;46.72\;{x_{3}}^{2}\;-\;233.58\;{x_{3}}^{1}\;+\;8315.3 \end{array}$$

(5) $$\begin{array}{c} \mathrm{RVS}\;\mathrm{at}\;100\;\mathrm{Hz}\\ =\;0.4252\;{x_{1}}^{2}\;{x_{2}}^{2}\;{x_{3}}^{2}\;-\;0.01816\;{x_{1}}^{2}\;{x_{2}}^{2}\;{x_{3}}^{1}\;-\;0.4365\;{x_{1}}^{2}\;{x_{2}}^{2}\;-\;0.4884\;{x_{1}}^{2}\;{x_{2}}^{1}\;{x_{3}}^{2}\;+\;0.002745\;{x_{1}}^{2}\;\\ \;{x_{2}}^{1}\;{x_{3}}^{1}\;+\;0.5230\;{x_{1}}^{2}\;{x_{2}}^{1}\;-\;0.3947\;{x_{1}}^{2}\;{x_{3}}^{2}\;+\;0.3745\;{x_{1}}^{2}\;{x_{3}}^{1}\;+\;0.1036\;{x_{1}}^{2}\;-\;0.4340\;{x_{1}}^{1}\;{x_{2}}^{2}\;{x_{3}}^{2}\;-\;\\ \;0.007676\;{x_{1}}^{1}\;{x_{2}}^{2}\;{x_{3}}^{1}\;+\;0.4671\;{x_{1}}^{1}\;{x_{2}}^{2}\;+\;0.4652\;{x_{1}}^{1}\;{x_{2}}^{1}\;{x_{3}}^{2}\;-\;0.01789\;{x_{1}}^{1}\;{x_{2}}^{1}\;{x_{3}}^{1}\;-\;0.5944\;{x_{1}}^{1}\;\\ \;{x_{2}}^{1}\;-\;0.4037\;{x_{1}}^{1}\;{x_{3}}^{2}\;+\;0.3699\;{x_{1}}^{1}\;{x_{3}}^{1}\;-\;1.917\;{x_{1}}^{1}\;+\;0.06814\;{x_{2}}^{2}\;{x_{3}}^{2}\;-\;0.02132\;{x_{2}}^{2}\;{x_{3}}^{1}\;+\;0.1046\;\\ \;{x_{2}}^{2}\;+\;0.04012\;{x_{2}}^{1}\;{x_{3}}^{2}\;-\;0.001864\;{x_{2}}^{1}\;{x_{3}}^{2}\;+\;0.6895\;{x_{2}}^{1}\;-\;0.1166\;{x_{3}}^{2}\;+\;1.646\;{x_{3}}^{1}\;-\;185.74 \end{array}$$

## 5. Comparison of the Flextensional Hydrophones of Different Classes

Figure 27 shows the RVS spectra of the flextensional hydrophones of all classes with the dimensions in Table 8. Figure 27 presents the response of the hydrophones from 10 Hz up to 10 kHz. The acoustic characteristics of the optimized hydrophone models of all of the classes are summarized in Table 9 to compare their performance at once. The class IV flextensional hydrophone turned out to have the widest bandwidth of 7.6 kHz. The ratio of the bandwidth to the peak RVS was defined as ‘fractional bandwidth’, and the fractional bandwidth of the class IV hydrophone was as high as 88.4%. In addition, the class IV hydrophone had the highest RVS at 100 Hz. The second class to have the widest bandwidth was found to be class III with a bandwidth of 6.9 kHz, which corresponded to the fractional bandwidth of 83.1%.

Finally, to confirm the superiority of the broadband class IV flextensional hydrophone designed in this study, the designed performance was compared with that of typical commercial hydrophones, i.e., the spherical hydrophone D/70/H and ring hydrophone T406 of Neptune (East Yorkshire, UK), as shown in Table 10 [5]. For comparison, we chose the bandwidth as the −3 dB frequency range, and the RVS level as the average sensitivity across the bandwidth. The class IV flextensional hydrophone showed a superior fractional bandwidth to and a competitive sensitivity with the commercial hydrophones. Therefore, the class IV flextensional hydrophone designed in this study is well suited as a high-sensitivity broadband hydrophone.

## 6. Conclusions

In this study, we developed low-frequency broadband flextensional hydrophones using the finite element method and optimal design technique. A comparison of the acoustic performance of the flextensional hydrophones optimized for each class revealed that the class IV flextensional hydrophone had the widest bandwidth and the highest RVS level at a low frequency, e.g., 100 Hz. The superiority of the class IV flextensional hydrophone was confirmed through a comparison of its performance with representative commercial hydrophones.

The most notable disadvantage of the class IV flextensional hydrophone is its complex structure compared to typical cylindrical or spherical hydrophones. Additionally, the class IV flextensional hydrophone requires a certain amount of pre-stress to account for the effects of water pressure during operation at certain depths. However, the superior performance of flextensional hydrophones justifies the complexity of their structure and the necessity of pre-stress.

The class IV flextensional hydrophone designed in this study is expected to find many applications in a wide ocean environment. Our future work includes manufacturing a prototype of the flextensional hydrophone and the experimental verification of the designed performance.

## Figures and Tables

**Table 1:** Properties of the materials constituting the flextensional hydrophones.

	Rubber	Aluminum	Steel
Density (kg/m^3^)	1100	2710	7500
Longitudinal velocity (m/s)	700	5850	6139
Shear velocity (m/s)	10	3127	3281

**Table 2:** Dimensions of the class I flextensional hydrophone basic model.

Structural Parameter	Dimension (mm)
Shell thickness ($t_{s1}$)	3.5
Flange radius ($r_{f1}$)	30.0
Radius of curvature of the shell (*ROC*)	450.0
Piezoceramic disk thickness ($t_{p1}$)	5.8
Outer radius of piezoceramic disk (${or}_{p1}$)	12.0
Inner radius of piezoceramic disk (${ir}_{p}$)	5.0

**Table 3:** Dimensions of the class III flextensional hydrophone basic model.

Structural Parameter	Dimension (mm)
Flange radius ($r_{f3}$)	35.0
Outer radius of piezoceramic disk (${or}_{p3}$)	20.0

**Table 4:** Dimensions of the class IV flextensional hydrophone basic model.

Structural Parameter	Dimension (mm)
Shell thickness ($t_{s4}$)	7.0
Shell height ($h_{s4}$)	27.0
Semi minor axis length ($l_{min}$)	15.0
Semi major axis length ($l_{maj}$)	32.5
Piezoceramic stack thickness ($t_{ps}$)	4.6
Insert width ($w_{i4}$)	6.0
Piezoceramic stack width ($w_{p}$)	4.6

**Table 5:** Dimensions of the class V flextensional hydrophone basic model.

Structural Parameter	Dimension (mm)
Cavity apex radius ($r_{ca5}$)	5.0
Cavity base radius ($r_{cb5}$)	23.0
Piezoceramic disk radius ($r_{p5}$)	30.0
Cavity height ($h_{c5}$)	2.0
Piezoceramic disk thickness ($t_{p5}$)	4.0
Shell thickness ($t_{s5}$)	3.0
Cavity apex radius ($r_{ca5}$)	5.0

**Table 6:** Dimensions of the class VI flextensional hydrophone basic model.

Structural Parameter	Dimension (mm)
Cavity base radius ($r_{cb6}$)	18.0
Cavity height ($h_{c6}$)	3.5
Shell thickness ($t_{s6}$)	2.0

**Table 7:** Dimensions of the class VII flextensional hydrophone basic model.

Structural Parameter	Dimension (mm)
Shell thickness ($t_{s7}$)	10.0
Shell height ($h_{s7}$)	40.0
Flange half-width (${hw}_{f}$)	40.0
Flange length ($l_{f}$)	21.0
Insert width ($w_{i7}$)	16.0

**Table 8:** Optimal dimension of the design variables for each class of flextensional hydrophones.

Class	Design Variable	Optimized Value (mm)
I	*t*_s_	2.8
	*r*_f_	32.9
	*or*_p_	14.4
III	*t*_s_	3.4
	*r*_f_	42.0
	*or*_p_	24.2
IV	*t*_s_	7.2
	*h*_s_	32.4
	*l*_min_	16.8
V	*t*_s_	3.6
	*r*_cb_	26.0
	*h*_c_	2.4
VI	*t*_s_	1.6
	*r*_cb_	18.4
	*h*_c_	3.9
VII	*t*_s_	9.9
	*h*_s_	40.0
	*l*_f_	25.0
	*w*_f_	33.5

**Table 9:** Acoustic characteristics of the optimized flextensional hydrophones of different classes.

	Class I	Class III	Class IV	Class V	Class VI	Class VII
Peak RVS frequency (kHz)	8.5	8.3	8.6	8.4	8.7	8.5
RVS at 100 Hz (dB)	−189.4	−194.3	−180.0	−190.9	−191.3	−189.1
Bandwidth (kHz)	5.7	6.9	7.6	4.8	5.0	6.2
Fractional bandwidth (%)	67.1	83.1	88.4	57.1	57.5	72.9

**Table 10:** Comparison of the acoustic characteristics of the class IV flextensional hydrophone and commercial hydrophones [5].

	Average RVS Level Across the Receive Bandwidth	−3 dB Fractional Bandwidth
D/70/H	−205 dB	62%
T406	−181 dB	30%
Class IV flextensional hydrophone	−179 dB	88%

## Data Availability

The original contributions presented in the study are included in the article, further inquiries can be directed to the corresponding author.
